# Implementation of a Bundle of Care to Reduce Surgical Site Infections in Patients Undergoing Vascular Surgery

**DOI:** 10.1371/journal.pone.0071566

**Published:** 2013-08-13

**Authors:** Jasper van der Slegt, Lijckle van der Laan, Eelco J. Veen, Yvonne Hendriks, Jannie Romme, Jan Kluytmans

**Affiliations:** 1 Department of Surgery, Amphia Hospital, Breda, The Netherlands; 2 Laboratory for Microbiology and Infection Control, Amphia Hospital, Breda, The Netherlands; 3 Department of Medical Microbiology and Infection Control, VU University Medical Center, Amsterdam, The Netherlands; University of Maryland, School of Medicine, United States of America

## Abstract

**Background:**

Surgical site infections (SSI’s) are associated with severe morbidity, mortality and increased health care costs in vascular surgery.

**Objective:**

To implement a bundle of care in vascular surgery and measure the effects on the overall and deep-SSI’s rates.

**Design:**

Prospective, quasi-experimental, cohort study.

**Methods:**

A prospective surveillance for SSI’s after vascular surgery was performed in the Amphia hospital in Breda, from 2009 through 2011. A bundle developed by the Dutch hospital patient safety program (DHPSP) was introduced in 2009. The elements of the bundle were (1) perioperative normothermia, (2) hair removal before surgery, (3) the use of perioperative antibiotic prophylaxis and (4) discipline in the operating room. Bundle compliance was measured every 3 months in a random sample of surgical procedures and this was used for feedback.

**Results:**

Bundle compliance improved significantly from an average of 10% in 2009 to 60% in 2011. In total, 720 vascular procedures were performed during the study period and 75 (10.4%) SSI were observed. Deep SSI occurred in 25 (3.5%) patients. Patients with SSI’s (28,5±29.3 vs 10.8±11.3, p<0.001) and deep-SSI’s (48.3±39.4 vs 11.4±11.8, p<0.001) had a significantly longer length of hospital stay after surgery than patients without an infection. A significantly higher mortality was observed in patients who developed a deep SSI (Adjusted OR: 2.96, 95% confidence interval 1.32–6.63). Multivariate analysis showed a significant and independent decrease of the SSI-rate over time that paralleled the introduction of the bundle. The SSI-rate was 51% lower in 2011 compared to 2009.

**Conclusion:**

The implementation of the bundle was associated with improved compliance over time and a 51% reduction of the SSI-rate in vascular procedures. The bundle did not require expensive or potentially harmful interventions and is therefore an important tool to improve patient safety and reduce SSI’s in patients undergoing vascular surgery.

## Introduction

The occurrence of surgical site infections (SSI’s) in surgery results in reduced quality of life, increased hospital length of stay, increased likelihood of mortality, higher change of readmissions and re-interventions [1]. In vascular surgery deep infections lead to re-operations with a high morbidity and even mortality, especially in patients who received a prosthetic graft resulting in higher health care costs [2], [3]. Therefore in 2007 a study was performed in the Netherlands to determine the amount of preventable mortality in Dutch hospitals [4]. SSI’s were amount the leading causes and based on these findings the Dutch hospital patient safety program (DHPSP) was developed. The DHPSP disseminates knowledge to the Dutch hospitals, especially in preventive programs and networking opportunities. Since the start in 2009, they set up a goal to reduce these highly preventable complications by 50% at the end of 2012. The program included a bundle to prevent the development of SSI (http://www.vmszorg.nl/10-Themas/POWI) and consisted of four process measures which should be implemented with a total compliance of at least 90%. To quantify the effect of the interventions on the outcome the SSI-rate was measured using validated methods in a national registry (). The bundle elements were: (1) perioperative normothermia, (2) appropriate hair removal before surgery, (3) the use of perioperative antibiotic prophylaxis and (4) discipline in the operation room. The first three measurements are evidence-based and are included in the current national guidelines for the prevention of SSI (http://www.wip.nl/free_content/Richtlijnen/100720powi%20def.pdf). The effect of improved discipline is generally recognized as an important aspect but few studies have addressed it. This is mainly caused by the lack of reliable methods to measure discipline. The expert team for SSI-prevention of the DHPSP decided to use the number of door openings during surgical procedures as a surrogate marker for discipline.

The objective of this study was to implement the bundle of care in vascular surgery and evaluate the effects on the overall SSI-rate as well as the deep SSI-rate while adjusting for confounders.

## Methods

A prospective surveillance study for SSI’s based on the criteria of the Centers for Disease Control [5] was performed in the Amphia hospital in Breda. This is a large teaching hospital located in the south of the Netherlands with 45.000 clinical admissions in 2011 and five consultant vascular surgeons. The hospital’s infection control committee and the board of directors as part of the patient safety program approved this study. Also the medical ethical committee of the Amphia Hospital in Breda, the Netherlands, permitted this project and waived informed consent.

Patients with a peripheral or central vascular reconstruction between March 2009 and January 2012 were included for analysis. The following characteristics were recorded: age, gender, ASA-score, length, weight, body mass index, wound class, type of procedure, central versus peripheral procedure, elective versus urgent, temperature at the end of surgery, duration of surgery, surgeon, number of vascular procedures per surgeon during the study period, admission date, date of surgery, discharge date, readmission within the post-discharge period, development of a SSI, development of a deep SSI and mortality within 6 months after the initial procedure.

Registration of SSÌs was performed by dedicated and specifically trained infection control personnel routinely performed the surveillance. Post-discharge surveillance was performed on all patients until 6 months after the date of the procedure.

The definition for SSI’s and deep-SSI’s, as described elsewhere, was based on the criteria of the Centers for Disease Control [5].

In 2009 the bundle to reduce SSI’s as defined by the DHPSP was implemented in our hospital. Starting in June 2009 bundle adherence was measured every three months using a random sample of 10 procedures. Normothermia was defined as a core temperature between 36.0°C and 38.0°C at the end of the surgical procedure. Perioperative prophylaxis was considered correct when the indicated antibiotic (according to the hospital formulary) was given between 15 and 60 minutes before the incision. Hair removal was preferably not performed and when it was done a clipper had to be used. Use of a razor blade was not allowed. Finally, the number of door-openings was measured from opening of the sterile equipment until the surgical wound was closed. This was done by visual inspection performed by infection control personnel. The target for door-openings was <10 per hour. Besides the amount of door-openings the reason for entering the operation room was also recorded. Eventually these data were used for feedback and development of strategies for improvement.

The development of the introduction of the bundle was evaluated after each measurement in a multidisciplinary team consisting of surgeons, anesthetists, the head of the operating room, operating room personnel and infection control personnel. Every three months the results of the adherence to the bundle was communicated to all personnel involved in the surgical process. They received a newsletter that informed them about the results of the bundle compliance and recommendations for improvement. The management of the hospital supported the program (which included more surgical specialties than vascular surgery) by the allocation of one full time equivalent infection control nurse to the program for surveillance of SSI, bundle measurements and feedback.

The following interventions for improvement were performed:

1. Hair removal was performed by a clipper instead of a razorblade
2. Timing and the use of pre-operative antibiotics were agreed upon by all participants and written in a protocol that could be handled by anesthesia personnel without consulting the surgeon. Before the operation started a time-out procedure was in place, which included the administration of antibiotic prophylaxis.
3. Temperature of the patient was measured during the entire process from the ward to the operating theatre and back to the ward. Based on the findings an isolation blanket was administered to patients on the ward before they were transported to the operating room. Previously this blanket had been administered in the operating theatre.
4. Door openings were subjected to a root-cause analysis. The multidisciplinary team critically assessed the determinants of openings and recommendations for improvement were made. The management of the OR was responsible for implementation of these recommendations. The main interventions were: reducing changes of the team for coffee breaks, making sure all equipment was present before the surgical procedure started and not entering the operating room for social talks during the surgical procedure.
5. For the implementation of the bundle a safety culture was promoted, in which personal feedback was done when the bundle adherence was at risk.
6. After each bundle measurement a newsletter as described before was provided as feedback about the progress of the introduction of the bundle.

### Statistical Analysis

All variables were univariate tested as appropriate by Fishers exact test or Students T-test. Variables with P<0.2 in univariate analysis were included in a multivariable logistic regression model. P<0.05 was considered statistically significant. Mortality was compared using Kaplan Meier survival analysis. A Cox-regression analysis was performed to adjust for confounders.

## Results

**Figure 1:**
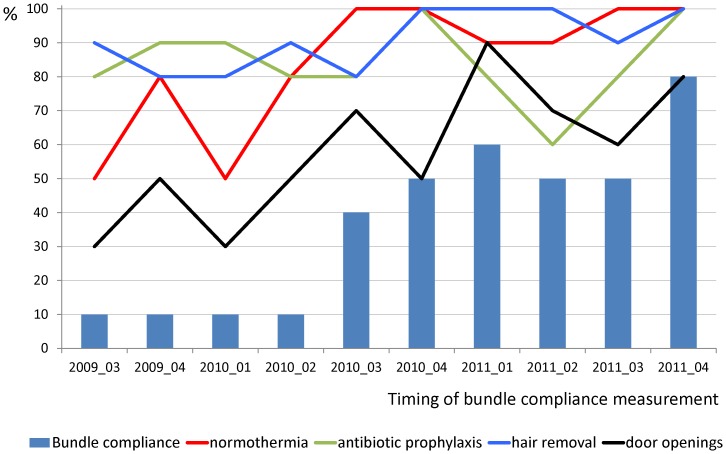
Compliance with the bundle and its individual components in repeated measurements from June 2009 through October 2011.

**Figure 2:**
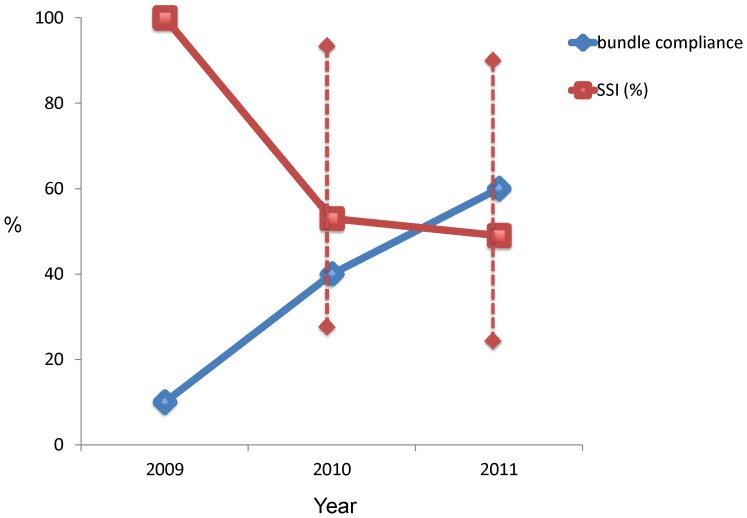
Annual changes in the surgical site infection (SSI) rate and bundle compliance and the 95% confidence interval.

Figure 1 shows that the compliance of the bundle per annual quarter started at 10% and improved to 80% by the end of 2011. At the start, the individual bundle elements had a variable compliance. Correct prophylactic antibiotic use was already high from the start of the program. Door movements were the most resilient topic for improvement (Figure 2). The annual compliance with the bundle elements increased gradually and statistically significant from 10% in 2009 to 60% in 2011 (Fig. 2, p<0.05).

### Surgical Site Infections

In a total of 720 vascular procedures 75 (10.4%) SSI’s were documented. The SSI-rate was significantly higher in peripheral versus central procedures (table 1). The SSI-rate declined significantly over time. Compared with 2009, the SSI-rate was 44% lower in 2011. Continuous variables in relation to the occurrence of SSI demonstrated that patients who developed a SSI had a significantly longer duration of the surgical procedure (Table 2).

**Table 1:** Categorical variables in relation to the occurrence of surgical site infections (SSI) after vascular surgery.

Determinant		DeepSSI/N	%	RR	95% CI	p-value
Gender	Male	54/538	10.0			
	Female	21/182	11.5	1.17	0.69–2.00	0.576
Procedure	Central	23/368	6.2			
	Peripheral	52/352	14.8	2.36	1.48–3.78	<0.001
Number of procedures per surgeon during study	1–100	27/229	11.8			
	>100	48/491	9.8	0.83	0.53–1.30	0.433
ASA class	1 or 2*	18/179	10.1			
	3,4 of 5	57/532	10.7	1.07	0.61–1.88	0.889
Wound score	1	73/700	10.4			
	≥2	2/20	10.0	0.95	0.22–4.20	1.000
Urgency of procedure	Elective	71/642	11.1			
	Non-elective	4/77	5.2	0.44	0.16–1.24	0.164
Death within 6 months	Yes	11/69	15.9			
	No	64/651	9.8	0.62	0.342–1.11	0.143
Year	2009	27/181	14.9			
	2010	27/290	9.3	0.62	0.35–1.10	0.074
	2011	21/249	8.4	0.57	0.31–1.03	0.043

ASA class: American Society of Anesthesiologists classification.

Wound class: Classification based on the intrinsic contamination of the incision site.

* ASA 1∶3 pts, ASA 2∶176 pts.

**Table 2:** Continuous variables in relation to the occurrence of surgical site infections (SSI) after vascular surgery.

	With SSI		Without SSI		
	mean	SD	Mean	SD	p-value
Age in years	68.9	11.5	70.8	9.8	0.129
Duration of surgery in minutes	168.0	74.5	146.4	65.6	0.008
Body mass index in kg/m^2^	26.1	5.0	25.6	4.0	0.367
Length of hospital stay aftersurgery	28.5	29.3	10.8	11.3	<0.001

A significant reduction of the SSI rate was observed in 2010 and 2011, with a 51% reduction in the last year of the study, compared to 2009. The increased compliance with the bundle was therefore associated with a decrease of the SSI-rate (Fig. 2).

### Deep Surgical Site Infections

In a total of 720 vascular procedures 25 (3.5%) deep-SSI’s were documented. This occurred significantly more in peripheral versus central procedures and the incidence in 2011 compared to the start of the study in 2009 was 44% lower (table 3 and 4).

**Table 3:** Categorical variables in relation to the occurrence of deep surgical site infections (deep SSI) after vascular surgery.

Determinant		DeepSSI/N	%	RR	95% CI	p-value
Gender	Male	17/538	3.2			
	Female	8/182	4.4	1.41	0.60–3.32	0.482
Procedure	Central	5/368	1.4			
	Peripheral	20/352	5.7	4.18	1.59–11.02	<0.001
Number of procedures per surgeon during study	1–100	12/229	5.2			
	>100	13/491	2.6	0.51	0.23–1.09	0.084
ASA class	1 or 2*	7/179	3.9			
	3,4 of 5	18/532	3.4	0.86	0.35–2.10	0.815
Wound score	1	23/700	3.3			
	≥2	2/20	10.0	3.27	0.72–14.94	0.150
Urgency of procedure	Elective	23/642	3.6			
	Non-elective	2/77	2.6	0.72	0.17–3.11	1.000
Death within 6 months	Yes	7/69	10.1			
	No	18/651	2.8	0.27	0.12–0.63	0.006
Year	2009	9/181	5.0			
	2010	9/290	3.1	0.62	0.24–1.60	0.33
	2011	7/249	2.8	0.57	0.21–1.55	0.27

ASA class: American Society of Anesthesiologists classification.

Wound class: Classification based on the intrinsic contamination of the incision site.

* ASA 1∶3 pts, ASA 2∶176 pts.

**Table 4:** Continuous variables in relation to the occurrence of deep surgical site infections (deep SSI) after vascular surgery.

	With SSI		Without SSI		
	mean	SD	Mean	SD	p-value
Age in years	72.0	10.9	70.5	9.9	0.472
Duration of surgery in minutes	150.9	53.1	148.6	67.3	0.861
Body mass index in kg/m^2^	25.5	4.9	25.7	4.1	0.764
Length of hospital stay aftersurgery	48.3	39.4	11.4	11.8	<0.001

To adjust for confounding a logistic regression analysis was performed as shown in table 5. With the exception of gender, age and elective versus non-elective procedures most variables that were identified in the univariate analysis retained their statistical significance.

**Table 5:** Multivariate analysis of variables in relation to the occurrence of surgical site infections (SSI) after vascular surgery with adjusted Odds ratio’s (AOR) and the 95% confidence interval (CI).

Variable	AOR	95% CI	p-value
Gender	1.09	0.63–1.91	0.753
Central versus peripheral procedure	0.40	0.23–0.68	0.001
Elective versus acute procedures	0.45	0.23–1.92	0.447
Duration of surgery (10 minutes)	1.04	1.00–1.00	0,022
age (years)	0.98	0.96–1.00	0.106
year (2010 versus 2009)	0.53	0.29–0.95	0.032
year (2011 versus 2009)	0.49	0.26–0.91	0.024

### Outcome in Patients After Surgical Site Infections

Patients who developed a SSI had a longer post-operative length of stay in the hospital (mean additional length of stay: 18 days (Table 2). In patients who developed deep SSI the post-operative length of stay was even longer (mean additional length of stay: 37 days (Table 4).

**Figure 3:**
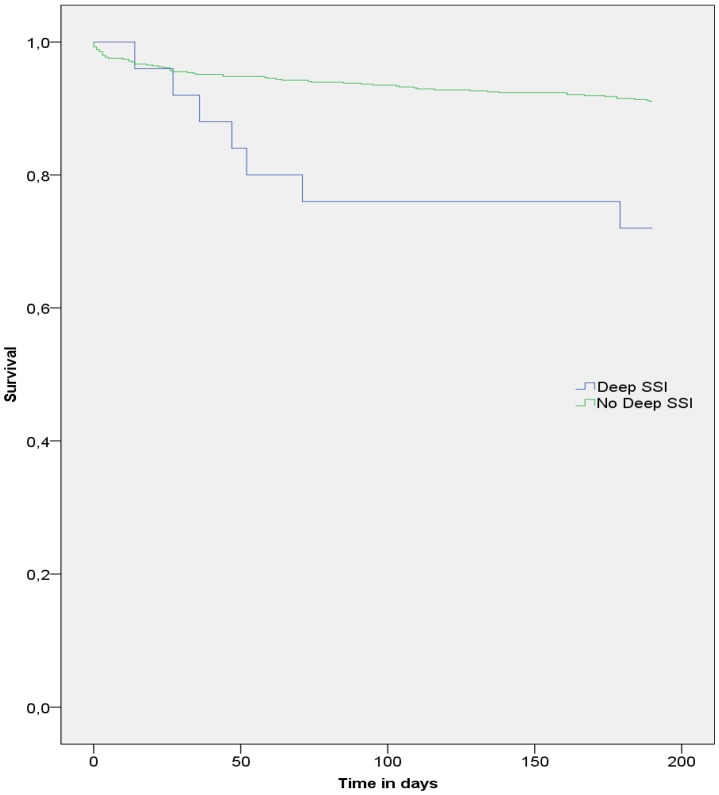
Kaplan-meier curve of 6 months survival in patients with and without a deep surgical site infection (deep SSI).

No statistical significant difference was seen in the Kaplan Meier curve for 6 months mortality of patients with and without a SSI (P:0.126 using the Log rank test). However, there was a significant difference in 6 months mortality of patients with and without deep SSI (P<0.001 (fig. 3)). Cox-regression analysis demonstrated a statistical significant difference for mortality between patients with and without deep SSI’s adjusted for ASA score and age (adjusted OR: 2.96, 95% confidence interval 1.32–6.6).

## Discussion

Our results confirm that SSI’s are relatively frequent (10.4%) and severe complications among patients who undergo vascular surgery. In this study, patients who developed a SSI had a prolonged hospital stay (18 days), which was even more pronounced when deep SSI occurred: mean length of hospital stay (37 days) and in these patients there was also a significantly higher mortality rate. This stresses the importance in preventing surgical site infections. The introduction of the bundle as defined by the DHPSP was associated with a strong and significant reduction of the SSI-rate in patients after vascular surgery. Due to the design of the study we cannot entirely be sure that there have been other unknown factors that contributed to the reduction of the SSI-rate. As we adjusted for confounders we consider it very likely that the reduction of the SSI-rate was largely due to the introduction of the bundle. The bundle exists of four components, which are scientifically proven and do not require expensive or complicated interventions. For vascular surgery, a 50% reduction would mean that approximately 18 SSI are prevented per year in our institute. Based on the additional length of stay of 18 days this would potentially save over 300 days of hospital stay per year. The mean costs a day in our hospital are estimated to be approximately €500, meaning that the total savings would be close to €150,000. Since the bundle elements are cheap it is likely that this is a highly cost-effective intervention. Our maximum bundle compliance per measurement was 80%. This is less than the 90% that was defined as a target at the onset of the program. Therefore, additional improvements are possible and may even further reduce the SSI-rate. We have continued the program and by the end of 2012 the compliance was 90%. As the SSI-rate can only be determined after a follow up of half a year the effects on the SSI-rate cannot be determined at this moment.

The importance of a bundle of care to reduce hospital infections have become clear since a large multicentre study was performed in the United States of America to reduce catheter-related-infections and in a later study the reduction of ventilator-associated pneumonia in the ICU [6], [7]. A few studies have described the effects of a bundle of measurements on the prevention of SSI’s after colorectal surgery. One study in colorectal surgery demonstrated a positive trend but had a small sample size so no definite conclusions could be drawn [8]. Another randomized study had a disappointing result, as the SSI-rate in the group with the bundle was increased [9] ^.^ The main criticism on this study is that the bundle used, was mainly based on technical adjustments and did not encourage a structural improvement in the overall surgical process to create a safety culture.

The bundle that we used has already been described before [10]. It was studied in the same time period in our hospital for the prevention of SSI’s after colorectal surgery [11]. Also after colorectal surgery a substantial (36%) and significant reduction of the SSI-rate was observed after implementation of the bundle. We observed an even stronger effect of the implementation of the bundle on the reduction of SSI’s in vascular surgery. Another study published in august 2012 also described a reduction of SSI’s in patients having colorectal surgery after the implementation of a comparable bundle of care. The following bundle of six measurements was introduced: standardization of skin preparation; administration of preoperative chlorhexidine showers; selective elimination of mechanical bowel preparation; warming of patients in the preanesthesia area; adoption of enhanced sterile techniques for skin and fascial closure; addressing previously unrecognized lapses in antibiotic prophylaxis [12]. After implementation, they observed a 33.3% reduction of SSI’s, which was comparable to the results with colorectal surgery in our institute. Due to their SSI’s reduction they also concluded that the implementation of the bundle resulted in major annual cost savings in their institution. This assumption is not entirely valid since it cannot be assumed that patients with SSI’s or deep SSI’s will not be suffering from other underlying reasons that cause their longer length of hospital stay. However it is likely that reducing SSI’s is associated with lower annual cost.

In our study, the wound care and compliance with hand hygiene during the postoperative phase could also contribute to the development of SSI’s and this was not included in the intervention. However, since we adhered to the national guidelines on woundcare (www.wip.nl) and only included primary closed wounds which are less prone to post-operative contamination and did not require special postoperative wound care we assume no important contributory effect of wound care on the development of SSI’s. The effects of hand hygiene compliance on the wards could have had a negative effect on the development of SSI’s and should not be underestimated. In this study, we did not analyze the hand hygiene compliance. A contributory effect can therefore not be excluded.

The longer length of hospital stay in patients with SSI’s is, as mentioned before, not necessarily caused by the SSI’s but may in part be due to other underlying factors. This study cannot define the contribution of SSI’s to the observed increase in the length of hospital stay. However, others have shown that SSI’s cause a substantial length of hospital stay and a double blind randomized study using a preventive intervention showed that prevention of SSI’s is associated with substantial cost savings [13]. Therefore, we conclude that SSI’s will have an important effect on the length of hospital stay but the exact amount cannot be determined in this study.

There are some other limitations to be mentioned to this study. First we could not demonstrate a direct causal relationship between the implementation of the bundle and the reduction of SSI since we did not use a randomized controlled study design. This study design is not feasible, when a change in behavior is part of the intervention. Health care workers cannot change their behavior based on individual randomization. Second, the year before the introduction of the bundle, our hospital was participating in the SURPASS study [14] that introduced the time-out procedure and the preoperative checklist. Since this study finished one year before we started this study, we cannot exclude a residual effect of this study on our results. Third, we did not perform an interrupted time series analysis since the interventions were implemented over a longer period of time (1 year and through the entire study period). Finally, we cannot exclude the positive effects of the routine feedback moments and discussions causing unknown behaviour effects on SSI reduction. They may have contributed to the reduction of SSI-rate as well.

In vascular surgery SSI’s have been reported to occur in 5–10% of patients (central and peripheral procedures) and could become life-threatening especially when prosthetic grafts are involved. This makes SSI-reduction an important topic for improvement. Mostly technical preventative measurements have been described to reduce vascular SSI’s. There are also studies suggesting the importance of maintaining peri-operative physiological parameters (oxygen, temperature, blood sugar and intravascular volumes) [2], [3]. To our knowledge none of these evidence based inventions has been bundled and structurally implemented to reduce surgical site infections in vascular surgery. To reduce these SSI’s, we implemented a bundle of measurements introduced by the DHPSP. Others and we demonstrated that the strict implementation of the bundle results in a substantial and significant reduction of the infection rate and therefore improves the safety for surgical patients having vascular procedures. As the bundle did not involve expensive or potentially harmful elements we recommend the implementation of this bundle on a large scale.
